# Supplementary material for: Genome-Wide Identification of Callose Synthase Family Genes and Their Expression Analysis in Floral Bud Development and Hormonal Responses in Prunus mume
Source: Plants (Basel). 2023 Dec 14;12(24):4159. doi: 10.3390/plants12244159 (PMC10748206; doi:10.3390/plants12244159)
Supplement: Supplementary file 1 [file plants-12-04159-s001.zip › plants-2743198-supplementary.pdf]

Figure S1. The featured motifs of PmGSL proteins.

Motif 1

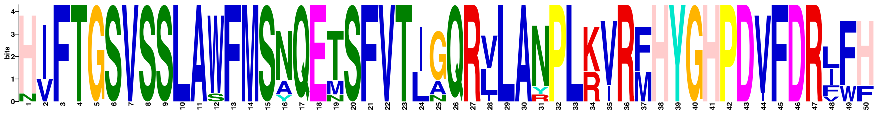

Motif 2

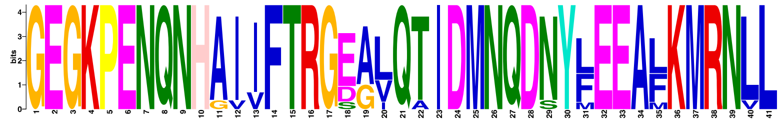

Motif 3

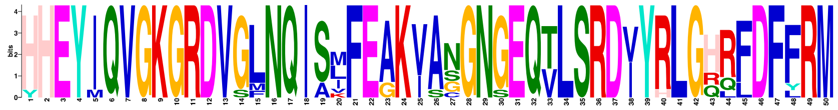

Motif 4

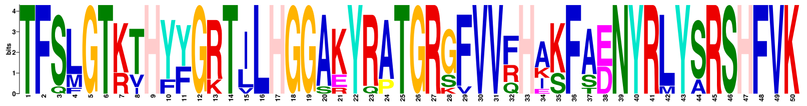

Motif 5

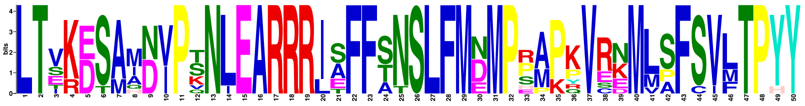

Figure S2. The sequence alignment within GSL domains for PmGSL proteins visualized with GeneDoc.

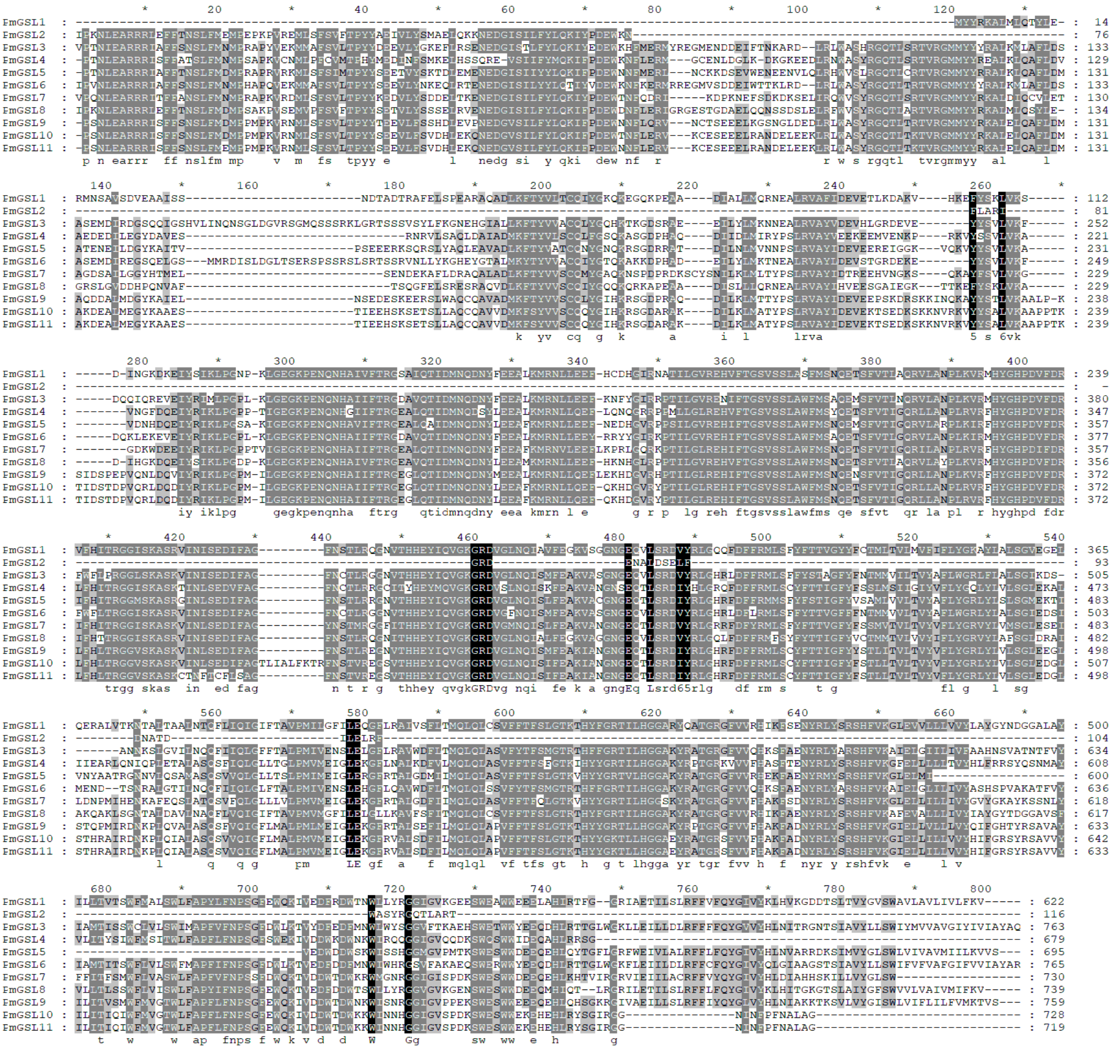

Figure S3. The chromosomal location of *PmGSL* genes.

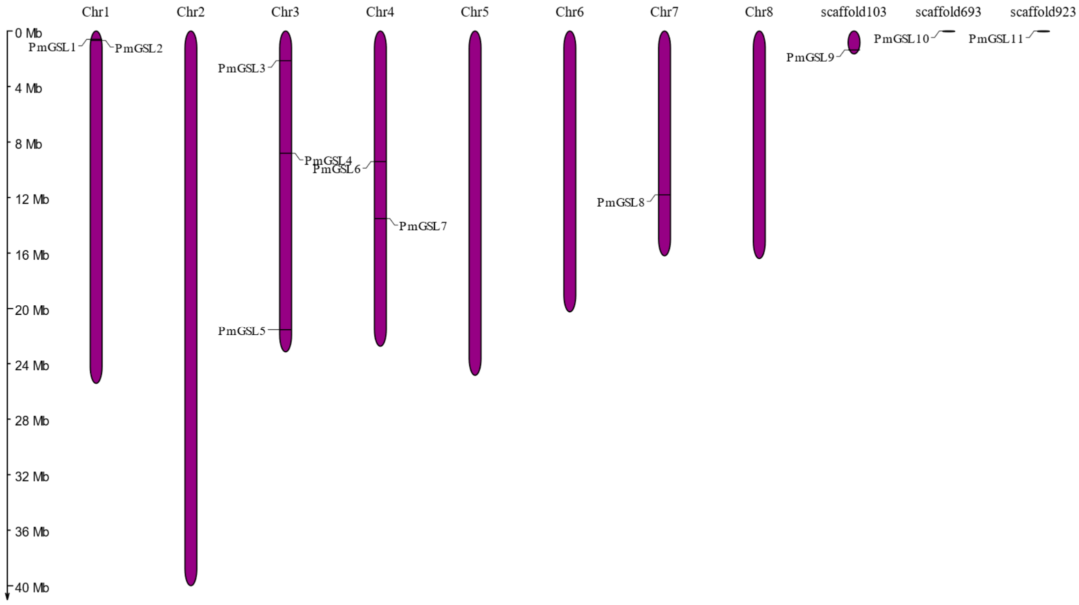

Figure S4. Selective pressure analysis of three *PmGSL* gene clades revealed strong purifying selection among codons. The residues colored with yellow or purple indicate signatures of positive or purifying selection, respectively.

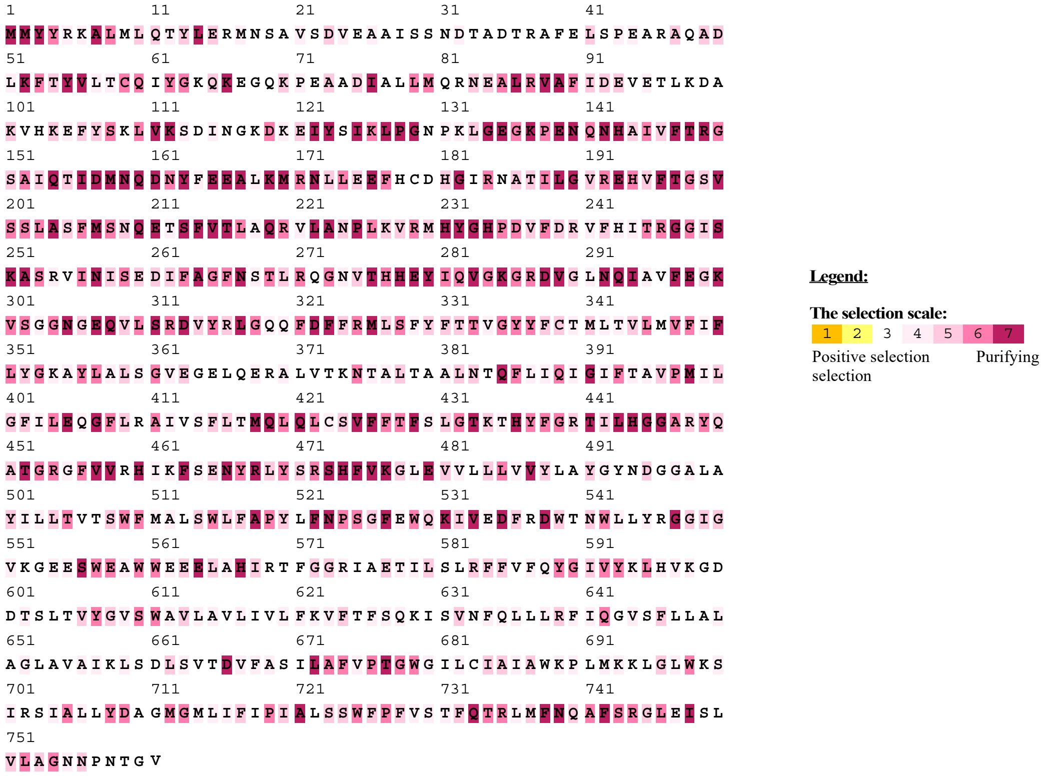

Table S1. Detailed information of GSL family genes identified in *Arabidopsis thaliana*, *Populus tricocarpa*, *Malus domestica*, *Prunus mume*, *Prunus persica*, *Oryza sativa* and *Sorghum bicolor*.

GeneID	Gene_name	NCBI accesion	Pfam accession	Pfam name	E-Value (Pfam)	Protein length (aa)	MW /Da	Chromosomal location	Subcellular prediction
AT1G05570.1	AtGSL6	NP_001322482.1	PF02364	Glucan_synthase	0.00E+00	1950	225761.03	Chr1	Plasma membrane
AT1G06490.1	AtGSL7	NP_001322002.1	PF02364	Glucan_synthase	0.00E+00	1958	228032.33	Chr1	Plasma membrane
AT2G13680.1	AtGSL2	NP_849953.2	PF02364	Glucan_synthase	0.00E+00	1923	220660.73	Chr2	Plasma membrane
AT2G31960.1	AtGSL3	NP_001323730.1	PF02364	Glucan_synthase	0.00E+00	1950	226019.49	Chr2	Plasma membrane
AT2G36850.1	AtGSL8	NP_850271.5	PF02364	Glucan_synthase	0.00E+00	1904	218378.13	Chr2	Plasma membrane
AT3G07160.1	AtGSL10	NP_187372.5	PF02364	Glucan_synthase	0.00E+00	1892	217379.53	Chr3	Plasma membrane
AT3G14570.1	AtGSL4	NP_001189893.1	PF02364	Glucan_synthase	0.00E+00	1976	228484.45	Chr3	Plasma membrane
AT3G59100.1	AtGSL11	NP_191469.3	PF02364	Glucan_synthase	0.00E+00	1921	223513.98	Chr3	Plasma membrane
AT4G03550.1	AtGSL5	NP_192264.1	PF02364	Glucan_synthase	0.00E+00	1780	206912.39	Chr4	Plasma membrane
AT4G04970.1	AtGSL1	NP_567278.1	PF02364	Glucan_synthase	0.00E+00	1768	205526.07	Chr4	Plasma membrane
AT5G13000.1	AtGSL12	NP_196804.6	PF02364	Glucan_synthase	0.00E+00	1955	226183.58	Chr5	Plasma membrane

AT5G36870.1	AtGSL9	NP_198503.3	PF02364	Glucan_synthase	0.00E+00	1871	216137.76	Chr5	Plasma membrane
Pm000093	PmGSL1	XP_034221744.1	PF02364	Glucan_synthase	0.00E+00	761	85842.58	Pm1	Plasma membrane
Pm000094	PmGSL2	KAI5321149.1	PF02364	Glucan_synthase	7.50E-38	1177	136609.85	Pm1	Plasma membrane
Pm010041	PmGSL3	XP_008225095.1	PF02364	Glucan_synthase	0.00E+00	1769	204837.86	Pm3	Plasma membrane
Pm011145	PmGSL4	XP_008226224.2	PF02364	Glucan_synthase	0.00E+00	1823	210775.28	Pm3	Plasma membrane
Pm012715	PmGSL5	XP_034210637.1	PF02364	Glucan_synthase	0.00E+00	1763	202350.42	Pm3	Plasma membrane
Pm014141	PmGSL6	CAB4273338.1	PF02364	Glucan_synthase	0.00E+00	1773	206642.53	Pm4	Plasma membrane
Pm014618	PmGSL7	XP_021811691.1	PF02364	Glucan_synthase	0.00E+00	1943	225407.81	Pm4	Plasma membrane
Pm024437	PmGSL8	XP_021813277.1	PF02364	Glucan_synthase	0.00E+00	1805	205401.5	Pm7	Plasma membrane
Pm028207	PmGSL9	TQE02010.1	PF02364	Glucan_synthase	0.00E+00	1970	227615.31	scaffold103	Plasma membrane
Pm030983	PmGSL10	XP_016652560.1	PF02364	Glucan_synthase	0.00E+00	1438	165118.44	scaffold693	Plasma membrane
Pm031333	PmGSL11	XP_050386959.1	PF02364	Glucan_synthase	0.00E+00	1873	215381.89	scaffold923	Plasma membrane
Prupe.2G003400.1	PpGSL1	KAB2594654.1	PF02364	Glucan_synthase	0.00E+00	1953	225298.36	Pp2	Plasma membrane

Prupe.3G138400.1	PpGSL2	CAB4273338.1	PF02364	Glucan_synthase	0.00E+00	1769	205957.77	Pp3	Plasma membrane
Prupe.3G175000.1	PpGSL3	XP_021811691.1	PF02364	Glucan_synthase	0.00E+00	1927	223680.75	Pp3	Plasma membrane
Prupe.4G047200.1	PpGSL4	XP_008225095.1	PF02364	Glucan_synthase	0.00E+00	1768	204446.3	Pp4	Plasma membrane
Prupe.4G152000.1	PpGSL5	KAH0986430.1	PF02364	Glucan_synthase	0.00E+00	1954	226500.7	Pp4	Plasma membrane
Prupe.4G271000.1	PpGSL6	XP_034210637.1	PF02364	Glucan_synthase	0.00E+00	1857	213342.48	Pp4	Plasma membrane
Prupe.5G158100.1	PpGSL7	CAB4280578.1	PF02364	Glucan_synthase	0.00E+00	1898	217307.09	Pp5	Plasma membrane
Prupe.6G014200.1	PpGSL8	XP_034221744.1	PF02364	Glucan_synthase	0.00E+00	1905	217857.96	Pp6	Plasma membrane
Prupe.6G014400.1	PpGSL9	TQE02010.1	PF02364	Glucan_synthase	0.00E+00	1957	225717.01	Pp6	Plasma membrane
MD01G1004100	MdGSL1	TQD78496.1	PF02364	Glucan_synthase	0.00E+00	1092	124996.34	Md1	Plasma membrane
MD02G1211500	MdGSL2	XP_034210637.1	PF02364	Glucan_synthase	0.00E+00	1924	220819.12	Md2	Plasma membrane
MD02G1319100	MdGSL3	KAB2594654.1	PF02364	Glucan_synthase	0.00E+00	1935	223298.76	Md2	Plasma membrane
MD03G1015000	MdGSL4	KAB2634296.1	PF02364	Glucan_synthase	0.00E+00	1959	226272.58	Md3	Plasma membrane
MD06G1068700	MdGSL5	XP_028960437.1	PF02364	Glucan_synthase	5.69E-20	220	24056.89	Md6	Plasma membrane

MD06G1158200	MdGSL6	XP_021813277.1	PF02364	Glucan_synthase	0.00E+00	1879	214582.97	Md6	Plasma membrane
MD07G1001800	MdGSL7	KAB2594654.1	PF02364	Glucan_synthase	6.05E-134	1411	163200.54	Md7	Plasma membrane
MD07G1002000	MdGSL8	TQD85101.1	PF02364	Glucan_synthase	2.16E-62	371	42688.4	Md7	Plasma membrane
MD09G1150100	MdGSL9	KAB2613297.1	PF02364	Glucan_synthase	1.72E-45	1220	142105.46	Md9	Plasma membrane
MD09G1150200	MdGSL10	TQD80962.1	PF02364	Glucan_synthase	0.00E+00	690	79402.04	Md9	Plasma membrane
MD09G1247400	MdGSL11	XP_004305416.1	PF02364	Glucan_synthase	0.00E+00	1769	205565.27	Md9	Plasma membrane
MD10G1295000	MdGSL12	KAB2601207.1	PF02364	Glucan_synthase	0.00E+00	1776	206251.68	Md10	Plasma membrane
MD11G1016100	MdGSL13	XP_034221744.1	PF02364	Glucan_synthase	0.00E+00	2009	230853.47	Md11	Plasma membrane
MD11G1016200	MdGSL14	XP_008338497.2	PF02364	Glucan_synthase	0.00E+00	1957	225949.16	Md11	Plasma membrane
MD11G1276200	MdGSL15	XP_048442946.1	PF02364	Glucan_synthase	0.00E+00	1952	226507.48	Md11	Plasma membrane
MD14G1164800	MdGSL16	TQD79031.1	PF02364	Glucan_synthase	0.00E+00	1965	225336.74	Md14	Plasma membrane
MD17G1137400	MdGSL17	XP_028953369.1	PF02364	Glucan_synthase	0.00E+00	1936	224402.04	Md17	Plasma membrane
MD17G1240300	MdGSL18	XP_004305416.1	PF02364	Glucan_synthase	0.00E+00	1620	188854.75	Md17	Plasma membrane

Potri.001G011900.2.p	PtGSL1	KAI5600220.1	PF02364	Glucan_synthase	0.00E+00	1905	218482.03	Pt1	Plasma membrane
Potri.001G012200.1.p	PtGSL2	KAG6790986.1	PF02364	Glucan_synthase	0.00E+00	1963	226047.46	Pt1	Plasma membrane
Potri.001G230000.4.p	PtGSL3	XP_034910185.1	PF02364	Glucan_synthase	0.00E+00	1949	225115.64	Pt1	Plasma membrane
Potri.002G058700.1.p	PtGSL4	TKS18072.1	PF02364	Glucan_synthase	0.00E+00	1941	224772.62	Pt2	Plasma membrane
Potri.003G214200.1.p	PtGSL5	KAI5596289.1	PF02364	Glucan_synthase	0.00E+00	1971	226881.25	Pt3	Plasma membrane
Potri.005G058300.1.p	PtGSL6	TKR75091.1	PF02364	Glucan_synthase	0.00E+00	1920	220573.94	Pt5	Plasma membrane
Potri.005G203500.2.p	PtGSL7	XP_011044776.1	PF02364	Glucan_synthase	0.00E+00	1931	223866.83	Pt5	Plasma membrane
Potri.011G052400.1.p	PtGSL8	KAG6754863.1	PF02364	Glucan_synthase	0.00E+00	1778	206035.7	Pt11	Plasma membrane
Potri.011G095100.2.p	PtGSL9	XP_024437439.1	PF02364	Glucan_synthase	0.00E+00	1947	224782.83	Pt11	Plasma membrane
Potri.013G131350.1.p	PtGSL10	XP_052302287.1	PF02364	Glucan_synthase	0.00E+00	1765	205572.14	Pt13	Plasma membrane
Potri.015G089300.1.p	PtGSL11	KAH8486790.1	PF02364	Glucan_synthase	0.00E+00	1898	217438.61	Pt15	Plasma membrane
LOC_Os01g34880.1	OsGSL1	XP_015613872.1	PF02364	Glucan_synthase	3.27E-107	496	57163.31	Os1	Plasma membrane
LOC_Os01g34890.1	OsGSL2	XP_052135104.1	PF02364	Glucan_synthase	6.52E-111	1224	143121.8	Os1	Plasma membrane

LOC_Os01g34930.1	OsGSL3	EEE54737.1	PF02364	Glucan_synthase	6.16E-117	560	64443.05	Os1	Plasma membrane
LOC_Os01g48200.1	OsGSL4	KAB8081731.1	PF02364	Glucan_synthase	2.49E-132	469	53760.87	Os1	Plasma membrane
LOC_Os01g55040.1	OsGSL5	EEE55159.1	PF02364	Glucan_synthase	0.00E+00	1790	207241.27	Os1	Nucleus
LOC_Os02g14900.1	OsGSL6	EEC71499.1	PF02364	Glucan_synthase	0.00E+00	1366	157989.96	Os2	Plasma membrane
LOC_Os02g58560.1	OsGSL7	XP_015626962.1	PF02364	Glucan_synthase	0.00E+00	894	103359.06	Os2	Plasma membrane
LOC_Os03g03610.1	OsGSL8	EEE58116.1	PF02364	Glucan_synthase	0.00E+00	1907	221235.51	Os3	Plasma membrane
LOC_Os06g02260.1	OsGSL9	EEC74436.1	PF02364	Glucan_synthase	3.93E-77	436	49559.47	Os6	Plasma membrane
LOC_Os06g08380.1	OsGSL10	BAD67750.1	PF02364	Glucan_synthase	0.00E+00	1919	218921.59	Os6	Plasma membrane
LOC_Os06g51270.1	OsGSL11	XP_006657366.2	PF02364	Glucan_synthase	0.00E+00	1845	212453.38	Os6	Plasma membrane
Sobic.001G521500.1.p	ScGSL1	OQU93328.1	PF02364	Glucan_synthase	0.00E+00	1954	226331.11	Sc1	Plasma membrane
Sobic.001G529600.1.p	ScGSL2	KAG0553099.1	PF02364	Glucan_synthase	0.00E+00	1910	218402.45	Sc1	Plasma membrane
Sobic.001G542450.1.p	ScGSL3	VAH99526.1	PF02364	Glucan_synthase	6.77E-51	299	34160.15	Sc1	Plasma membrane
Sobic.001G542500.2.p	ScGSL4	KXG40423.1	PF02364	Glucan_synthase	2.25E-161	1419	163115.66	Sc1	Plasma membrane

Sobic.003G179600.1.p	ScGSL5	ONM41810.1	PF02364	Glucan_synthase	0.00E+00	1934	224136.35	Sc3	Plasma membrane
Sobic.003G180100.1.p	ScGSL6	ONM41655.1	PF02364	Glucan_synthase	0.00E+00	1839	212620.55	Sc3	Plasma membrane
Sobic.003G252500.1.p	ScGSL7	KAG0538854.1	PF02364	Glucan_synthase	0.00E+00	1779	205550.2	Sc3	Plasma membrane
Sobic.003G298900.1.p	ScGSL8	ONM37957.1	PF02364	Glucan_synthase	0.00E+00	1790	207649.41	Sc3	Nucleus
Sobic.004G107800.1.p	ScGSL9	XP_021314199.1	PF02364	Glucan_synthase	0.00E+00	1992	228905.52	Sc4	Plasma membrane
Sobic.004G358400.1.p	ScGSL10	NP_001385201.1	PF02364	Glucan_synthase	0.00E+00	1952	225015.11	Sc4	Plasma membrane
Sobic.010G064200.1.p	ScGSL11	KAF8732991.1	PF02364	Glucan_synthase	0.00E+00	1905	219104.4	Sc10	Plasma membrane
Sobic.010G275800.1.p	ScGSL12	AQK80739.1	PF02364	Glucan_synthase	0.00E+00	1965	226621.45	Sc10	Plasma membrane

Table S2. The duplication mode of GSL family genes from *Arabidopsis thaliana*, *Populus tricocarpa*, *Malus domestica*, *Prunus mume*, *Prunus persica*, *Oryza sativa* and *Sorghum bicolor.*

Gene_name	Duplication type
AtGSL6	WGD/Segmental
AtGSL7	Dispersed
AtGSL2	Dispersed
AtGSL3	WGD/Segmental
AtGSL8	Dispersed
AtGSL10	Dispersed
AtGSL4	Tandem
AtGSL11	Dispersed
AtGSL5	WGD/Segmental
AtGSL1	WGD/Segmental
AtGSL12	Tandem
AtGSL9	Dispersed
PmGSL1	Dispersed
PmGSL2	Dispersed
PmGSL3	Dispersed
PmGSL4	Dispersed
PmGSL5	Dispersed
PmGSL6	Dispersed
PmGSL7	Dispersed
PmGSL8	WGD/Segmental
PmGSL9	Dispersed
PmGSL10	Dispersed
PmGSL11	Dispersed
PpGSL1	Tandem
PpGSL2	Dispersed
PpGSL3	Tandem
PpGSL4	Dispersed
PpGSL5	Dispersed
PpGSL6	Dispersed
PpGSL7	Tandem
PpGSL8	Tandem
PpGSL9	Tandem
MdGSL1	Dispersed
MdGSL2	Dispersed
MdGSL3	WGD/Segmental
MdGSL4	WGD/Segmental
MdGSL5	Dispersed
MdGSL6	WGD/Segmental
MdGSL7	WGD/Segmental
MdGSL8	Dispersed

MdGSL9	WGD/Segmental
MdGSL10	Dispersed
MdGSL11	WGD/Segmental
MdGSL12	WGD/Segmental
MdGSL13	WGD/Segmental
MdGSL14	WGD/Segmental
MdGSL15	Dispersed
MdGSL16	Tandem
MdGSL17	WGD/Segmental
MdGSL18	WGD/Segmental
PtGSL1	Tandem
PtGSL2	Tandem
PtGSL3	Tandem
PtGSL4	Tandem
PtGSL5	Proximal
PtGSL6	Dispersed
PtGSL7	Dispersed
PtGSL8	Tandem
PtGSL9	Dispersed
PtGSL11	Tandem
OsGSL1	Proximal
OsGSL2	Dispersed
OsGSL3	Proximal
OsGSL4	WGD/Segmental
OsGSL5	WGD/Segmental
OsGSL6	Dispersed
OsGSL7	Tandem
OsGSL8	Tandem
OsGSL9	Tandem
OsGSL10	Dispersed
OsGSL11	Dispersed
ScGSL1	Dispersed
ScGSL2	Dispersed
ScGSL3	Dispersed
ScGSL4	Dispersed
ScGSL5	Proximal
ScGSL6	Proximal
ScGSL7	Tandem
ScGSL8	Tandem
ScGSL9	Tandem
ScGSL10	WGD/Segmental
ScGSL11	Tandem
ScGSL12	WGD/Segmental

Table S3. The cis-regulatory elements predicted for *PmGSL* genes and their detailed information.

Gene	Site Name	Organism	Position	Strand	Sequence	Function
PmGSL1	CAT-box	Arabidopsis thaliana	500	-	GCCACT	cis-acting regulatory element related to meristem expression
PmGSL1	CAT-box	Arabidopsis thaliana	1416	-	GCCACT	cis-acting regulatory element related to meristem expression
PmGSL1	CGTCA-motif	Hordeum vulgare	45	-	CGTCA	cis-acting regulatory element involved in the MeJA-responsiveness
PmGSL1	CGTCA-motif	Hordeum vulgare	107	-	CGTCA	cis-acting regulatory element involved in the MeJA-responsiveness
PmGSL1	GT1-motif	Arabidopsis thaliana	1703	+	GGTTAA	light responsive element
PmGSL1	GT1-motif	Arabidopsis thaliana	1797	+	GGTTAA	light responsive element
PmGSL1	I-box	Zea mays	159	+	gGATAAGGTG	part of a light responsive element
PmGSL1	I-box	Zea mays	996	+	cGATAAGGCG	part of a light responsive element
PmGSL1	LTR	Hordeum vulgare	920	+	CCGAAA	cis-acting element involved in low-temperature responsiveness
PmGSL1	LTR	Hordeum vulgare	1099	+	CCGAAA	cis-acting element involved in low-temperature responsiveness
PmGSL1	MYB	Arabidopsis thaliana	55	-	CAACAG	MYB binding site
PmGSL1	MYB	Arabidopsis thaliana	362	-	CAACCA	MYB binding site
PmGSL1	MYB	Arabidopsis thaliana	1438	-	TAACCA	MYB binding site

PmGSL1	MYB	Arabidopsis thaliana	1595	+	TAACCA	MYB binding site
PmGSL1	MYB	Arabidopsis thaliana	1702	-	TAACCA	MYB binding site
PmGSL1	MYB	Arabidopsis thaliana	1752	-	CAACCA	MYB binding site
PmGSL1	MYB	Arabidopsis thaliana	1756	-	TAACCA	MYB binding site
PmGSL1	MYB	Arabidopsis thaliana	1796	-	TAACCA	MYB binding site
PmGSL1	MYB	Arabidopsis thaliana	1833	-	TAACCA	MYB binding site
PmGSL1	MYC	Arabidopsis thaliana	358	-	CATTTG	MYC binding site
PmGSL1	MYC	Arabidopsis thaliana	435	-	CATTTG	MYC binding site
PmGSL1	MYC	Arabidopsis thaliana	1925	+	CATGTG	MYC binding site
PmGSL1	P-box	Oryza sativa	1203	+	CCTTTTG	gibberellin-responsive element
PmGSL1	TATC-box	Oryza sativa	1268	-	TATCCCA	cis-acting element involved in gibberellin-responsiveness
PmGSL1	TATC-box	Oryza sativa	1334	+	TATCCCA	cis-acting element involved in gibberellin-responsiveness
PmGSL1	TCA-element	Brassica oleracea	205	+	TCAGAAGAGG	cis-acting element involved in salicylic acid responsiveness

PmGSL2	ABRE	Arabidopsis thaliana	479	-	ACGTG	cis-acting element involved in the abscisic acid responsiveness
PmGSL2	ABRE	Arabidopsis thaliana	1075	-	ACGTG	cis-acting element involved in the abscisic acid responsiveness
PmGSL2	ABRE	Arabidopsis thaliana	1122	+	AACCCGG	cis-acting element involved in the abscisic acid responsiveness
PmGSL2	ABRE	Arabidopsis thaliana	1770	-	CACGTG	cis-acting element involved in the abscisic acid responsiveness
PmGSL2	ABRE	Arabidopsis thaliana	1771	+	ACGTG	cis-acting element involved in the abscisic acid responsiveness
PmGSL2	CGTCA-motif	Hordeum vulgare	14	+	CGTCA	cis-acting regulatory element involved in the MeJA-responsiveness
PmGSL2	CGTCA-motif	Hordeum vulgare	829	+	CGTCA	cis-acting regulatory element involved in the MeJA-responsiveness
PmGSL2	GA-motif	Arabidopsis thaliana	1205	-	ATAGATAA	part of a light responsive element
PmGSL2	G-box	Zea mays	1075	+	CACGTC	cis-acting regulatory element involved in light responsiveness
PmGSL2	G-box	Arabidopsis thaliana	1770	-	CACGTG	cis-acting regulatory element involved in light responsiveness
PmGSL2	GT1-motif	Arabidopsis thaliana	121	-	GGTTAA	light responsive element
PmGSL2	GT1-motif	Arabidopsis thaliana	396	-	GGTTAA	light responsive element
PmGSL2	MBS	Arabidopsis thaliana	488	-	CAACTG	MYB binding site involved in drought-inducibility

PmGSL2	MBS	Arabidopsis thaliana	1233	+	CAACTG	MYB binding site involved in drought-inducibility
PmGSL2	MYB	Arabidopsis thaliana	47	+	TAACCA	MYB binding site
PmGSL2	MYB	Arabidopsis thaliana	122	+	TAACCA	MYB binding site
PmGSL2	MYB	Arabidopsis thaliana	149	+	CAACCA	MYB binding site
PmGSL2	MYB	Arabidopsis thaliana	397	+	TAACCA	MYB binding site
PmGSL2	MYB	Arabidopsis thaliana	488	-	CAACTG	MYB binding site
PmGSL2	MYB	Arabidopsis thaliana	774	-	TAACCA	MYB binding site
PmGSL2	MYB	Arabidopsis thaliana	1218	-	CAACCA	MYB binding site
PmGSL2	MYB	Arabidopsis thaliana	1233	+	CAACTG	MYB binding site
PmGSL2	MYB	Arabidopsis thaliana	1482	+	TAACCA	MYB binding site
PmGSL2	MYB	Arabidopsis thaliana	1535	-	CAACCA	MYB binding site
PmGSL2	MYC	Arabidopsis thaliana	444	-	CATGTG	MYC binding site
PmGSL2	MYC	Arabidopsis thaliana	721	+	CAATTG	MYC binding site

PmGSL2	MYC	Arabidopsis thaliana	1183	+	CATTTG	MYC binding site
PmGSL2	MYC	Arabidopsis thaliana	1857	+	CATTTG	MYC binding site
PmGSL2	MYC	Arabidopsis thaliana	1958	+	CATTTG	MYC binding site
PmGSL2	TCA-element	Nicotiana tabacum	793	-	CCATCTTTTT	cis-acting element involved in salicylic acid responsiveness
PmGSL2	W box	Arabidopsis thaliana	889	+	TTGACC	cis-element for WRKYs
PmGSL3	ABRE	Arabidopsis thaliana	1626	+	ACGTG	cis-acting element involved in the abscisic acid responsiveness
PmGSL3	AE-box	Arabidopsis thaliana	955	+	AGAAACAA	part of a module for light response
PmGSL3	Box 4	Petroselinum crispum	651	+	ATTAAT	part of a conserved DNA module involved in light responsiveness
PmGSL3	Box 4	Petroselinum crispum	1095	-	ATTAAT	part of a conserved DNA module involved in light responsiveness
PmGSL3	Box 4	Petroselinum crispum	1116	-	ATTAAT	part of a conserved DNA module involved in light responsiveness
PmGSL3	Box 4	Petroselinum crispum	1144	-	ATTAAT	part of a conserved DNA module involved in light responsiveness
PmGSL3	Box 4	Petroselinum crispum	1221	-	ATTAAT	part of a conserved DNA module involved in light responsiveness
PmGSL3	CAG-motif	Arabidopsis thaliana	850	-	GAAAGGCAGAC	part of a light response element

PmGSL3	GARE-motif	Brassica oleracea	919	-	TCTGTTG	gibberellin-responsive element
PmGSL3	G-box	Zea mays	758	-	CACGAC	cis-acting regulatory element involved in light responsiveness
PmGSL3	GT1-motif	Arabidopsis thaliana	538	+	GGTTAA	light responsive element
PmGSL3	LTR	Hordeum vulgare	950	+	CCGAAA	cis-acting element involved in low-temperature responsiveness
PmGSL3	LTR	Hordeum vulgare	1610	-	CCGAAA	cis-acting element involved in low-temperature responsiveness
PmGSL3	MSA-like	Catharanthus roseus	598	-	(T/C)C(T/C)AACGG(T/C)(T/C)A	cis-acting element involved in cell cycle regulation
PmGSL3	MYB	Arabidopsis thaliana	549	+	TAACCA	MYB binding site
PmGSL3	MYB	Arabidopsis thaliana	656	-	TAACCA	MYB binding site
PmGSL3	MYB	Arabidopsis thaliana	919	+	CAACAG	MYB binding site
PmGSL3	MYB	Arabidopsis thaliana	1915	+	CAACCA	MYB binding site
PmGSL3	MYC	Arabidopsis thaliana	468	-	CATTTG	MYC binding site
PmGSL3	MYC	Arabidopsis thaliana	529	+	CATTTG	MYC binding site
PmGSL3	MYC	Arabidopsis thaliana	912	-	CATGTG	MYC binding site
PmGSL3	MYC	Arabidopsis thaliana	1290	-	CATGTG	MYC binding site

PmGSL3	MYC	Arabidopsis thaliana	1416	+	CATTTG	MYC binding site
PmGSL3	MYC	Arabidopsis thaliana	1538	+	CATGTG	MYC binding site
PmGSL3	P-box	Oryza sativa	358	+	CCTTTTG	gibberellin-responsive element
PmGSL3	P-box	Oryza sativa	943	-	CCTTTTG	gibberellin-responsive element
PmGSL3	TGA-element	Brassica oleracea	933	+	AACGAC	auxin-responsive element
PmGSL3	TGA-element	Brassica oleracea	1606	-	AACGAC	auxin-responsive element
PmGSL4	ABRE	Arabidopsis thaliana	946	+	ACGTG	cis-acting element involved in the abscisic acid responsiveness
PmGSL4	ABRE	Arabidopsis thaliana	1126	-	ACGTG	cis-acting element involved in the abscisic acid responsiveness
PmGSL4	ABRE	Arabidopsis thaliana	1667	-	CACGTG	cis-acting element involved in the abscisic acid responsiveness
PmGSL4	ABRE	Arabidopsis thaliana	1668	+	ACGTG	cis-acting element involved in the abscisic acid responsiveness
PmGSL4	AE-box	Arabidopsis thaliana	1337	-	AGAAACTT	part of a module for light response
PmGSL4	Box 4	Petroselinum crispum	1112	-	ATTAAT	part of a conserved DNA module involved in light responsiveness
PmGSL4	Box 4	Petroselinum crispum	1618	-	ATTAAT	part of a conserved DNA module involved in light responsiveness
PmGSL4	CAT-box	Arabidopsis thaliana	414	+	GCCACT	cis-acting regulatory element related to meristem expression

PmGSL4	CGTCA-motif	Hordeum vulgare	944	-	CGTCA	cis-acting regulatory element involved in the MeJA-responsiveness
PmGSL4	G-box	Zea mays	945	-	CACGTC	cis-acting regulatory element involved in light responsiveness
PmGSL4	G-box	Brassica napus	1666	-	ACACGTGT	cis-acting regulatory element involved in light responsiveness
PmGSL4	G-box	Arabidopsis thaliana	1667	-	CACGTG	cis-acting regulatory element involved in light responsiveness
PmGSL4	MBS	Arabidopsis thaliana	662	+	CAACTG	MYB binding site involved in drought-inducibility
PmGSL4	MBS	Arabidopsis thaliana	1071	-	CAACTG	MYB binding site involved in drought-inducibility
PmGSL4	MYB	Arabidopsis thaliana	151	+	CAACCA	MYB binding site
PmGSL4	MYB	Arabidopsis thaliana	662	+	CAACTG	MYB binding site
PmGSL4	MYB	Arabidopsis thaliana	1071	-	CAACTG	MYB binding site
PmGSL4	MYB	Arabidopsis thaliana	1289	-	CAACCA	MYB binding site
PmGSL4	MYC	Arabidopsis thaliana	1484	-	CATTTG	MYC binding site
PmGSL4	MYC	Arabidopsis thaliana	1517	+	CATGTG	MYC binding site
PmGSL4	MYC	Arabidopsis thaliana	1605	+	CATTTG	MYC binding site
PmGSL4	P-box	Oryza sativa	393	+	CCTTTTG	gibberellin-responsive element
PmGSL4	P-box	Oryza sativa	1983	+	CCTTTTG	gibberellin-responsive element

PmGSL4	TATC-box	Oryza sativa	1145	-	TATCCCA	cis-acting element involved in gibberellin-responsiveness
PmGSL4	TGA-element	Brassica oleracea	1853	+	AACGAC	auxin-responsive element
PmGSL4	W box	Arabidopsis thaliana	397	+	TTGACC	cis-element for WRKYs
PmGSL5	ABRE	Arabidopsis thaliana	1035	+	ACGTG	cis-acting element involved in the abscisic acid responsiveness
PmGSL5	ABRE	Triticum aestivum	1526	-	GACACGTGGC	cis-acting element involved in the abscisic acid responsiveness
PmGSL5	ABRE	Arabidopsis thaliana	1528	-	CACGTG	cis-acting element involved in the abscisic acid responsiveness
PmGSL5	ABRE	Arabidopsis thaliana	1529	+	ACGTG	cis-acting element involved in the abscisic acid responsiveness
PmGSL5	Box 4	Petroselinum crispum	893	+	ATTAAT	part of a conserved DNA module involved in light responsiveness
PmGSL5	Box 4	Petroselinum crispum	1111	-	ATTAAT	part of a conserved DNA module involved in light responsiveness
PmGSL5	Box 4	Petroselinum crispum	1989	-	ATTAAT	part of a conserved DNA module involved in light responsiveness
PmGSL5	GATA-motif	Arabidopsis thaliana	902	-	AAGATAAGATT	part of a light responsive element
PmGSL5	G-box	Arabidopsis thaliana	1034	+	TACGTG	cis-acting regulatory element involved in light responsiveness
PmGSL5	G-box	Brassica napus	1526	-	ACACGTGGC	cis-acting regulatory element involved in light responsiveness

PmGSL5	G-box	Arabidopsis thaliana	1528	-	CACGTG	cis-acting regulatory element involved in light responsiveness
PmGSL5	GT1-motif	Arabidopsis thaliana	949	-	GGTTAA	light responsive element
PmGSL5	GT1-motif	Arabidopsis thaliana	1377	-	GGTTAA	light responsive element
PmGSL5	HD-Zip 1	Arabidopsis thaliana	153	+	CAAT(A/T)ATTG	element involved in differentiation of the palisade mesophyll cells
PmGSL5	LTR	Hordeum vulgare	1395	+	CCGAAA	cis-acting element involved in low-temperature responsiveness
PmGSL5	LTR	Hordeum vulgare	1791	+	CCGAAA	cis-acting element involved in low-temperature responsiveness
PmGSL5	MBS	Arabidopsis thaliana	920	+	CAACTG	MYB binding site involved in drought-inducibility
PmGSL5	MSA-like	Catharanthus roseus	950	-	(T/C)C(T/C)AACGG(T/C)(T/C)A	cis-acting element involved in cell cycle regulation
PmGSL5	MYB	Arabidopsis thaliana	400	-	TAACCA	MYB binding site
PmGSL5	MYB	Arabidopsis thaliana	920	+	CAACTG	MYB binding site
PmGSL5	MYB	Arabidopsis thaliana	1020	-	TAACCA	MYB binding site
PmGSL5	MYB	Arabidopsis thaliana	1378	+	TAACCA	MYB binding site
PmGSL5	MYB	Arabidopsis thaliana	1744	+	CAACCA	MYB binding site
PmGSL5	MYC	Arabidopsis thaliana	1162	-	CATTTG	MYC binding site

PmGSL6	ABRE	Arabidopsis thaliana	1834	-	ACGTG	cis-acting element involved in the abscisic acid responsiveness
PmGSL6	AE-box	Arabidopsis thaliana	1641	+	AGAAACAA	part of a module for light response
PmGSL6	ATCT-motif	Pisum sativum	1258	+	AATCTAATCC	part of a conserved DNA module involved in light responsiveness
PmGSL6	Box 4	Petroselinum crispum	696	+	ATTAAT	part of a conserved DNA module involved in light responsiveness
PmGSL6	Box 4	Petroselinum crispum	1205	-	ATTAAT	part of a conserved DNA module involved in light responsiveness
PmGSL6	Box 4	Petroselinum crispum	1461	-	ATTAAT	part of a conserved DNA module involved in light responsiveness
PmGSL6	CAT-box	Arabidopsis thaliana	228	-	GCCACT	cis-acting regulatory element related to meristem expression
PmGSL6	CAT-box	Arabidopsis thaliana	235	-	GCCACT	cis-acting regulatory element related to meristem expression
PmGSL6	CGTCA-motif	Hordeum vulgare	290	-	CGTCA	cis-acting regulatory element involved in the MeJA-responsiveness
PmGSL6	CGTCA-motif	Hordeum vulgare	432	-	CGTCA	cis-acting regulatory element involved in the MeJA-responsiveness
PmGSL6	CGTCA-motif	Hordeum vulgare	1036	+	CGTCA	cis-acting regulatory element involved in the MeJA-responsiveness
PmGSL6	CGTCA-motif	Hordeum vulgare	1078	+	CGTCA	cis-acting regulatory element involved in the MeJA-responsiveness
PmGSL6	CGTCA-motif	Hordeum vulgare	1298	+	CGTCA	cis-acting regulatory element involved in the MeJA-responsiveness

PmGSL6	GT1-motif	Arabidopsis thaliana	1701	+	GGTTAA	light responsive element
PmGSL6	I-box	Zea mays	919	-	gGATAAGGTG	part of a light responsive element
PmGSL6	LTR	Hordeum vulgare	1784	+	CCGAAA	cis-acting element involved in low-temperature responsiveness
PmGSL6	MRE	Petroselinum crispum	1101	-	AACCTAA	MYB binding site involved in light responsiveness
PmGSL6	MSA-like	Catharanthus roseus	729	-	(T/C)C(T/C)AACGG(T/C)(T/C)A	cis-acting element involved in cell cycle regulation
PmGSL6	MYB	Arabidopsis thaliana	1700	-	TAACCA	MYB binding site
PmGSL6	MYC	Arabidopsis thaliana	324	-	CATGTG	MYC binding site
PmGSL6	MYC	Arabidopsis thaliana	347	-	CATGTG	MYC binding site
PmGSL6	MYC	Arabidopsis thaliana	799	-	CATTTG	MYC binding site
PmGSL6	MYC	Arabidopsis thaliana	1225	+	CATTTG	MYC binding site
PmGSL6	MYC	Arabidopsis thaliana	1237	-	CAATTG	MYC binding site
PmGSL6	MYC	Arabidopsis thaliana	1555	-	CATGTG	MYC binding site
PmGSL6	MYC	Arabidopsis thaliana	1557	+	CATGTG	MYC binding site
PmGSL6	TGA-element	Brassica oleracea	619	+	AACGAC	auxin-responsive element

PmGSL6	TGA-element	Brassica oleracea	1768	+	AACGAC	auxin-responsive element
PmGSL6	W box	Arabidopsis thaliana	437	+	TTGACC	cis-element for WRKYs
PmGSL6	W box	Arabidopsis thaliana	674	+	TTGACC	cis-element for WRKYs
PmGSL7	ABRE	Arabidopsis thaliana	1504	-	CACGTG	cis-acting element involved in the abscisic acid responsiveness
PmGSL7	ABRE	Arabidopsis thaliana	1505	+	ACGTG	cis-acting element involved in the abscisic acid responsiveness
PmGSL7	ABRE	Triticum aestivum	1544	-	GACACGTGGC	cis-acting element involved in the abscisic acid responsiveness
PmGSL7	ABRE	Arabidopsis thaliana	1546	-	CACGTG	cis-acting element involved in the abscisic acid responsiveness
PmGSL7	ABRE	Arabidopsis thaliana	1547	+	ACGTG	cis-acting element involved in the abscisic acid responsiveness
PmGSL7	AE-box	Arabidopsis thaliana	731	+	AGAAACTT	part of a module for light response
PmGSL7	Box 4	Petroselinum crispum	666	+	ATTAAT	part of a conserved DNA module involved in light responsiveness
PmGSL7	CAG-motif	Arabidopsis thaliana	119	+	GAAAGGCAGAC	part of a light response element
PmGSL7	CGTCA-motif	Hordeum vulgare	426	-	CGTCA	cis-acting regulatory element involved in the MeJA-responsiveness
PmGSL7	CGTCA-motif	Hordeum vulgare	1794	+	CGTCA	cis-acting regulatory element involved in the MeJA-responsiveness

PmGSL7	G-box	Arabidopsis thaliana	1504	-	CACGTG	cis-acting regulatory element involved in light responsiveness
PmGSL7	G-box	Arabidopsis thaliana	1544	-	GCCACGTGGA	cis-acting regulatory element involved in light responsiveness
PmGSL7	G-box	Arabidopsis thaliana	1546	-	CACGTG	cis-acting regulatory element involved in light responsiveness
PmGSL7	GT1-motif	Arabidopsis thaliana	1221	+	GGTTAA	light responsive element
PmGSL7	LTR	Hordeum vulgare	830	+	CCGAAA	cis-acting element involved in low-temperature responsiveness
PmGSL7	LTR	Hordeum vulgare	1993	-	CCGAAA	cis-acting element involved in low-temperature responsiveness
PmGSL7	MBS	Arabidopsis thaliana	385	+	CAACTG	MYB binding site involved in drought-inducibility
PmGSL7	MBS	Arabidopsis thaliana	1531	-	CAACTG	MYB binding site involved in drought-inducibility
PmGSL7	MRE	Petroselinum crispum	709	-	AACCTAA	MYB binding site involved in light responsiveness
PmGSL7	MYB	Arabidopsis thaliana	385	+	CAACTG	MYB binding site
PmGSL7	MYB	Arabidopsis thaliana	1157	-	TAACCA	MYB binding site
PmGSL7	MYB	Arabidopsis thaliana	1381	+	CAACCA	MYB binding site
PmGSL7	MYB	Arabidopsis thaliana	1531	-	CAACTG	MYB binding site
PmGSL7	MYB	Arabidopsis thaliana	1615	-	CAACAG	MYB binding site

PmGSL7	MYC	Arabidopsis thaliana	174	-	CATTTG	MYC binding site
PmGSL7	MYC	Arabidopsis thaliana	258	+	CATTTG	MYC binding site
PmGSL7	MYC	Arabidopsis thaliana	1153	-	CATGTG	MYC binding site
PmGSL7	MYC	Arabidopsis thaliana	1738	-	CATTTG	MYC binding site
PmGSL7	MYC	Arabidopsis thaliana	1926	+	CATTTG	MYC binding site
PmGSL7	P-box	Oryza sativa	1671	-	CCTTTTG	gibberellin-responsive element
PmGSL7	TCA-element	Nicotiana tabacum	1909	-	CCATCTTTTT	cis-acting element involved in salicylic acid responsiveness
PmGSL8	Box 4	Petroselinum crispum	1404	-	ATTAAT	part of a conserved DNA module involved in light responsiveness
PmGSL8	CGTCA-motif	Hordeum vulgare	57	-	CGTCA	cis-acting regulatory element involved in the MeJA-responsiveness
PmGSL8	circadian	Lycopersicon esculentum	366	-	CAAAGATATC	cis-acting regulatory element involved in circadian control
PmGSL8	MBS	Arabidopsis thaliana	1247	-	CAACTG	MYB binding site involved in drought-inducibility
PmGSL8	MRE	Petroselinum crispum	1619	-	AACCTAA	MYB binding site involved in light responsiveness
PmGSL8	MYB	Arabidopsis thaliana	1247	-	CAACTG	MYB binding site

PmGSL8	MYB	Arabidopsis thaliana	1313	+	CAACCA	MYB binding site
PmGSL8	MYB	Arabidopsis thaliana	1496	+	CAACCA	MYB binding site
PmGSL8	MYC	Arabidopsis thaliana	956	+	CATTTG	MYC binding site
PmGSL8	MYC	Arabidopsis thaliana	1169	+	CATTTG	MYC binding site
PmGSL8	P-box	Oryza sativa	1779	-	CCTTTTG	gibberellin-responsive element
PmGSL8	TCA-element	Brassica oleracea	770	+	TCAGAAGAGG	cis-acting element involved in salicylic acid responsiveness
PmGSL8	TGA-element	Brassica oleracea	546	+	AACGAC	auxin-responsive element
PmGSL8	TGA-element	Brassica oleracea	627	-	AACGAC	auxin-responsive element
PmGSL8	W box	Arabidopsis thaliana	694	+	TTGACC	cis-element for WRKYs
PmGSL8	W box	Arabidopsis thaliana	778	-	TTGACC	cis-element for WRKYs
PmGSL8	W box	Arabidopsis thaliana	961	-	TTGACC	cis-element for WRKYs
PmGSL9	ABRE	Arabidopsis thaliana	320	+	ACGTG	cis-acting element involved in the abscisic acid responsiveness
PmGSL9	ABRE	Arabidopsis thaliana	325	-	TACGGTC	cis-acting element involved in the abscisic acid responsiveness

PmGSL9	AE-box	Arabidopsis thaliana	1492	-	AGAAACTT	part of a module for light response
PmGSL9	Box 4	Petroselinum crispum	627	+	ATTAAT	part of a conserved DNA module involved in light responsiveness
PmGSL9	CGTCA-motif	Hordeum vulgare	318	-	CGTCA	cis-acting regulatory element involved in the MeJA-responsiveness
PmGSL9	CGTCA-motif	Hordeum vulgare	851	-	CGTCA	cis-acting regulatory element involved in the MeJA-responsiveness
PmGSL9	GARE-motif	Brassica oleracea	1703	+	TCTGTTG	gibberellin-responsive element
PmGSL9	GATA-motif	Arabidopsis thaliana	1983	+	GATAGGA	part of a light responsive element
PmGSL9	G-box	Zea mays	319	-	CACGTC	cis-acting regulatory element involved in light responsiveness
PmGSL9	G-box	Zea mays	438	-	CACGAC	cis-acting regulatory element involved in light responsiveness
PmGSL9	GT1-motif	Arabidopsis thaliana	1821	-	GGTTAA	light responsive element
PmGSL9	MYB	Arabidopsis thaliana	42	-	CAACCA	MYB binding site
PmGSL9	MYB	Arabidopsis thaliana	1704	-	CAACAG	MYB binding site
PmGSL9	MYC	Arabidopsis thaliana	314	+	CATGTG	MYC binding site
PmGSL9	MYC	Arabidopsis thaliana	1356	-	CATGTG	MYC binding site
PmGSL9	P-box	Oryza sativa	1427	-	CCTTTTG	gibberellin-responsive element

PmGSL9	TCA-element	Nicotiana tabacum	1093	-	CCATCTTTTT	cis-acting element involved in salicylic acid responsiveness
PmGSL9	W box	Arabidopsis thaliana	682	+	TTGACC	cis-element for WRKYs
PmGSL9	W box	Arabidopsis thaliana	902	+	TTGACC	cis-element for WRKYs
PmGSL10	CAT-box	Arabidopsis thaliana	237	-	GCCACT	cis-acting regulatory element related to meristem expression
PmGSL10	CAT-box	Arabidopsis thaliana	283	+	GCCACT	cis-acting regulatory element related to meristem expression
PmGSL10	CAT-box	Arabidopsis thaliana	346	-	GCCACT	cis-acting regulatory element related to meristem expression
PmGSL10	CGTCA-motif	Hordeum vulgare	516	-	CGTCA	cis-acting regulatory element involved in the MeJA-responsiveness
PmGSL10	GARE-motif	Brassica oleracea	258	+	TCTGTTG	gibberellin-responsive element
PmGSL10	GT1-motif	Avena sativa	739	-	GGTTAAT	light responsive element
PmGSL10	GT1-motif	Arabidopsis thaliana	740	-	GGTTAA	light responsive element
PmGSL10	MYB	Arabidopsis thaliana	259	-	CAACAG	MYB binding site
PmGSL10	MYB	Arabidopsis thaliana	613	+	CAACAG	MYB binding site
PmGSL10	MYB	Arabidopsis thaliana	741	+	TAACCA	MYB binding site

PmGSL10	W box	Arabidopsis thaliana	747	-	TTGACC	cis-element for WRKYs
PmGSL11	ABRE	Arabidopsis thaliana	791	+	ACGTG	cis-acting element involved in the abscisic acid responsiveness
PmGSL11	AE-box	Arabidopsis thaliana	775	+	AGAAACTT	part of a module for light response
PmGSL11	Box 4	Petroselinum crispum	255	+	ATTAAT	part of a conserved DNA module involved in light responsiveness
PmGSL11	CGTCA-motif	Hordeum vulgare	146	+	CGTCA	cis-acting regulatory element involved in the MeJA-responsiveness
PmGSL11	CGTCA-motif	Hordeum vulgare	400	-	CGTCA	cis-acting regulatory element involved in the MeJA-responsiveness
PmGSL11	CGTCA-motif	Hordeum vulgare	422	-	CGTCA	cis-acting regulatory element involved in the MeJA-responsiveness
PmGSL11	CGTCA-motif	Hordeum vulgare	789	-	CGTCA	cis-acting regulatory element involved in the MeJA-responsiveness
PmGSL11	GARE-motif	Brassica oleracea	1718	+	TCTGTTG	gibberellin-responsive element
PmGSL11	G-box	Zea mays	790	-	CACGTC	cis-acting regulatory element involved in light responsiveness
PmGSL11	GT1-motif	Arabidopsis thaliana	1831	+	GGTTAA	light responsive element
PmGSL11	LTR	Hordeum vulgare	1618	+	CCGAAA	cis-acting element involved in low-temperature responsiveness
PmGSL11	MBS	Arabidopsis thaliana	1182	+	CAACTG	MYB binding site involved in drought-inducibility

PmGSL11	MYB	Arabidopsis thaliana	88	-	TAACCA	MYB binding site
PmGSL11	MYB	Arabidopsis thaliana	1182	+	CAACTG	MYB binding site
PmGSL11	MYB	Arabidopsis thaliana	1719	-	CAACAG	MYB binding site
PmGSL11	MYB	Arabidopsis thaliana	1943	+	CAACAG	MYB binding site
PmGSL11	MYC	Arabidopsis thaliana	621	-	CATGTG	MYC binding site
PmGSL11	MYC	Arabidopsis thaliana	623	+	CATGTG	MYC binding site
PmGSL11	MYC	Arabidopsis thaliana	1091	+	CATGTG	MYC binding site
PmGSL11	P-box	Oryza sativa	83	+	CCTTTTG	gibberellin-responsive element
PmGSL11	TCA-element	Nicotiana tabacum	665	+	CCATCTTTTT	cis-acting element involved in salicylic acid responsiveness
PmGSL11	TCA-element	Nicotiana tabacum	1214	+	CCATCTTTTT	cis-acting element involved in salicylic acid responsiveness
PmGSL11	TGA-element	Brassica oleracea	105	-	AACGAC	auxin-responsive element
PmGSL11	W box	Arabidopsis thaliana	900	+	TTGACC	cis-element for WRKYs

Table S4. The primers of *PmGSL* genes used in the qRT-PCR assays.

Gene	Primer	Sequence
PmGSL1	Pm000093-F	TCACTGCTGTCCCGATGATT
	Pm000093-R	GACAACAAATCCCCTGCCTG
PmGSL2	Pm000094-F	GTGTGGGGATTCTTGCTTGG
	Pm000094-R	GACCAGCATCCACCTTGTTC
PmGSL3	Pm010041-F	TTGCTACCACCCCGAAGAAT
	Pm010041-R	CCTGATCGTCCCTTCTCTCC
PmGSL4	Pm011145-F	TCATGCCTGAGTGTCTGTGT
	Pm011145-R	CAGCTGTCCCACTTTTGCTT
PmGSL5	Pm012715-F	CTGAGCGAGGACATCTTTGC
	Pm012715-R	AAGAAGTCAAAACGGTGCCC
PmGSL6	Pm014141-F	TCCTTGCTTGATGTGGGGAT
	Pm014141-R	CACCACACGCTTATTTGCCT
PmGSL7	Pm014618-F	GGATGTAATGGTCAACGGCC
	Pm014618-R	CCTGCGAGCTTCCAAGTTTT
PmGSL8	Pm024437-F	TGGTGGTGCTGTTTCTTTCG
	Pm024437-R	CCCACCAAGATTCCCAGCTA
PmGSL9	Pm028207-F	CATGCCAGAGTGCCTTTGTT
	Pm028207-R	TCGGCTTCCTTTGCAATCAC
PmGSL10	Pm030983-F	CAATGGGGCAATAGTCTTGTCTT
	Pm030983-R	TCCAAAATCCCAACTCCCTGA
PmGSL11	Pm031333-F	ACGGGTGAGCACATAAAACC
	Pm031333-R	CGACTGGCTGGCAAAAGAAA
